# Validation of rubric‐based evaluation for bachelor's theses in a food science and technology degree

**DOI:** 10.1111/1750-3841.17044

**Published:** 2024-04-05

**Authors:** Ana I. Andrés, María L. Timón, Juan Morillo, Concepción Marín, Juan Florencio Tejeda, Concepción Ayuso

**Affiliations:** ^1^ School of Agricultural Engineering University of Extremadura Badajoz Spain

**Keywords:** bachelor's thesis, peer evaluation, rubrics, validation

## Abstract

**Abstract:**

An effective evaluation of a bachelor's thesis (BT) needs the use of valid tools such as rubrics. There are few studies providing a validation of rubrics for these theses and even fewer in the academic field of food science and technology; hence the aim of this study was to validate a rubric for the assessment of relevant competencies in the BT. Ninety‐seven students presented their thesis and 56 teachers participated as members of the committees. The degree of relevance, suitability, and clarity of the rubrics were assessed as well as the relationship between student perception and academic performance and the congruence between the teacher's and students’ evaluations. The content validity index and intraclass correlation coefficient (ICC) were calculated. Students had a moderate level of knowledge about rubrics, but they mostly agreed with the premise that the rubrics are an accurate tool to assess the quality of BTs. Teacher's and student's marks mostly aligned. No significant relationship was found between the positive perception of rubric's validity and the final grades. Regarding clarity, suitability, and relevance as perceived by teachers, the used rubrics were highly valued. The ICC of criteria indicated that the rubrics were valid in the studied terms. Hence, the validation of a food science and technology degree BT assessment system based on a rubric has been achieved. Supporting Information

**Practical Application:**

The conclusions drawn from this research could enable teachers at other universities to use this methodology for validating their rubrics for bachelor's theses. Furthermore, they could use this validated rubric to evaluate projects presented at their respective institutions.

## 1 INTRODUCTION

The bachelor's thesis (BT) is a work designed to demonstrate the skills acquired by the students throughout the entire degree program and to verify the achievement of the global learning objectives. Moreover, it shows the level of maturity and professionalism reached in a degree study, which is directed by a tutor or a teacher. Once all the credits required for a degree have been fulfilled, a BT must be drafted and publicly defended to obtain the corresponding accreditation (Espinosa, 2014). The BT, a consequence of what has been learned throughout the studies, can be of different typologies: laboratory research, bibliographic review, or work of professional nature. The BT involves developing a project that includes explanations, theories, ideas, reasoning, and evaluations on a particular subject. As students work on this project, they develop a range of skills, including planning, analytical thinking, logical reasoning, problem‐solving, decision‐making, interpretation, communication, and evaluation. These skills are likely to be valuable in their future careers (Abas & Imam, 2016).

Evaluation committees assess the final work and the oral defense, considering how the theories on the subject have been applied, which methodology has been considered and why; what practical proposals have been derived from the research; and what have been the conclusive and critical reflections. When evaluators give a mark to the BT, what they really do is to judge a series of skills and knowledge. The evaluation is a complex process as it must be fair, objective, continuous, and formative (Alcarria et al., 2018). In this sense, a current trend considers rubrics as one of the most recommended instruments for the evaluation of competencies and skills. The interest on rubrics from an academic research approach is increasing over the years (Panadero et al., 2023). Rubrics are a powerful instrument for the evaluation of any type of task as they allow dividing the global tasks into simpler ones. They are defined as qualitative descriptors that establish the level of achievement of students in specific tasks, from the least acceptable to the maximum resolution. They translate the level of achievement to a score, improving the reliability and validating the evaluation, as students and teachers know what is evaluated (Jönsson & Panadero, 2017) by levels of skill acquisition, promoting the self‐evaluation of students and the feedback to them (Ferrer‐Pardo et al., 2022). Thus, a rubric could be an instrument to facilitate the assessment of the BT as it provides indicators for a precise evaluation of both final work and oral defense. As a matter of fact, most universities in Spain provide public rubrics for assessing BTs.

To be effective, rubrics must be valid, reliable, and fair. The validity refers to the accuracy and relevance with which an instrument measures what it is intended to be measured, and reliability refers to the level of consistency or agreement of the results when the same instrument is applied to the same subjects on different occasions (González‐Chordá et al., 2016). Several studies have addressed the analysis of reliability, reproducibility, and consistency of the rubric by different methods (García‐Ros, 2011), but studies on specific academic fields should be performed to use the rubric as a consistent evaluation tool (Panadero et al., 2023). In addition, reticence about the use of this type of evaluation for student assessing is considerable as their use requires some practice, knowledge, and effort (Ito, 2015).

In a review about the reliability, validity, and effectiveness of rubrics in higher education, the author concluded that the variety of methods used is wide, including expert judgment, factor analysis, questionnaires, indicating that none of them was preferable to another for this purpose (Brookhart, 2018). In this sense, Allen and Knight (2009) described the steps of a method for collaboratively developing and validating a rubric that integrates descriptors suggested by academics and professionals. In another study, García‐Ros (2011) analyzed the convergent validity of a rubric to evaluate oral communication skills of university students carried out by teachers and by peers. The level of convergence between the assessments made by the teachers and by the peers was analyzed from an analytical perspective (descriptor by descriptor) and holistic (global score). This author also analyzed the perception of validity and usefulness of the rubric from the assessments made by the students. On the other hand, Tobón et al. (2021) validated a rubric to evaluate pedagogical practices in teachers. The authors concluded that the rubric had a validity of content and construct, as well as reliability for the studied group. In other studies, Saito et al. (2021) proposed the Goal Question Metric (GQM) method to evaluate the characteristics of a rubric to assess programing‐thinking skills, confirming the generality of these traits, and the consistency and the validity of the GQM method. Sánchez‐Ramírez et al. (2022) designed and validated a rubric to evaluate competencies for employability. Expert judgment validated contents quantitatively and qualitatively (content validation). Lastly, Ferrer‐Pardo et al. (2022) demonstrated how the use of a rubric for oral presentation was a good instrument for a fair and consistent evaluation, by studying student's perception of the usefulness of the rubric related to their academic marks and analyzing the correlation between marks given by teachers and students when the rubric was used. Nevertheless, to the best of our knowledge, no scientific studies have approached the validation of rubrics of BTs in the field of food science and technology.

Therefore, the objective of this study was to validate an assessment rubric for relevant competencies in the BT in Food Science and Technology Degree at the Agricultural Engineering School of the University of Extremadura (UEX). For this purpose, the degree of relevance, suitability, and clarity of the designed rubrics have been analyzed as well as the relationship between student perception of the rubric and their academic performance and the congruence between the teacher's and students’ evaluations.

## 2 MATERIALS AND METHODS

### 2.1 Contextualization and design of the experiment

The study was carried out during the 2022–2023 academic year, for the evaluation of the BT presented by students in February, June, July, and September calls of the 2022/2023 academic year. BTs are a 6‐ECTS (European Credit Transfer and Accumulation System) course and consists of the preparation of a final written report and a dissertation about a topic related to the degree in Food Science and Technology. BT is supervised by one or two teachers of the degree and evaluated, both report and oral dissertation, by a committee composed of three teachers with expertise in the topic. Only laboratory research BT has been considered in this study.

### 2.2 Design of the rubric

The criteria for these rubrics were developed by the BT School Commission, consisting of nine experts, each one a member of the main teaching departments related to the Food Science and Technology Degree. The official competencies for this degree at the UEX were also considered for the development of the rubrics (https://www.unex.es/conoce‐la‐uex/centros/eia/titulaciones/info/competencias?=0516). Valid Assessment of Learning in Undergraduate Education (VALUE) rubrics (https://www.aacu.org/value‐rubrics), developed by the Association of American Colleges and Universities (Rhodes, 2010), was also taken into consideration by the committee. In addition, the BT committee developed the first version of the rubrics with ongoing inquiries to the rest of the faculty members during the 2021/2022 academic year.

The designed rubrics (Table 1) establish the criteria to be considered for the evaluation of the written BT report and its oral defense. The rubric includes 6 criteria and 11 subcriteria with 4‐score ratings (“Very appropriate,” “Appropriate,” “To improve,” and “Inadequate”). All items on the rubric used for BT evaluation are listed in Appendix A.

**TABLE 1:** Example of rubrics.

A. Written expression and written presentation of the work (15%)				
	Very appropriate	To improve	Appropriate	Inadequate
**Organization and structure (score from 0 to 10)**	Organization and structure are clear and suitable	Organization and structure are clear and adequate, but some nonessential sections are missing	Organization and structure are clear and adequate, but some essential sections are missing	Organization and structure are incoherent and disjointed. Essential parts of the work are missing
**Writing (score from 0 to 10)**	Coherent and correct writing, with no spelling mistakes or grammatical errors	Coherent and correct writing for the most part, with no spelling mistakes or grammatical errors	Inconsistent and incorrect wording. No spelling mistakes or grammatical errors	Inconsistent and incorrect writing, with spelling mistakes and/or grammatical errors
**Diagrams, tables, graphs (score from 0 to 10)**	Complies exactly with published drafting standards	Complies with most of the published drafting standards	Complies with some published drafting standards	Does not conform to published drafting standards
**Objectives (score from 0 to 10)**	Objectives are clear and appropriate, written in the infinitive form	Objectives are adequate and written in the infinitive although they could have been made more explicit	Objectives are not adequate, although they are well written and explained	Objectives are not adequate, neither well written nor explained

All the teachers evaluated the BTs using the developed rubrics as tool. These rubrics were also available for students during the whole course and were encouraged to get familiarized with them, through oral communication at class, Moodle or through social media.

### 2.3 Validation of the rubric

#### 2.3.1 Participants

Ninety‐seven students presented their BT at different calls of the year, 54.97% were female, and 45.03% male, with an age ranging between 22 and 26 years. Fifty‐six teachers, of which 44.6% were female and 55.4% were male, participated as members of the committees to evaluate the BT. This research was performed in accordance with the local ethical guidelines of the UEX and the ethical principles of the Helsinki Declaration.

#### 2.3.2 Validation process

After the oral defense, the members of the evaluating committee proceeded to give their marks of the BT presented by the corresponding students, according to their skill acquisition using the BT rubrics mentioned in Section 2.2. Teachers filled in a rubric excel questionnaire (Appendix B). In the same document, teachers were also requested to provide a numeric qualification based on their own criteria, without using the rubric as a tool. Teachers were advised to do this before using the rubric.

In addition, after the oral defense, the students were requested to complete a Google questionnaire (Google Forms, Google) regarding their perception of the BT rubrics and their level of agreement with the final grade assigned by the teacher in comparison to their self‐assessment. The students answered to this questionnaire on a 5‐point quantitative Likert scale, where 1 is understood as “Strongly Disagree” and 5 is considered “Strongly Agree” with the items of the questionnaire. In the same questionnaire, students were requested to assess the percentage of congruence between self‐assessment (self‐qualifications) and the academic grades assigned by the committee (“less than 20%,” “between 20% and 40%,” “between 40% and 60%,” “between 60% and 80%,” “between 80% and 100%”). They were also asked to provide the final grade they would have assigned to themselves. Data were collected anonymously, and gender was not considered.

Finally, teachers were also asked to make a quantitative evaluation of each one of the rubric indicators established to assess the final mark of the BT: the degree of clarity in the wording, the suitability of the indicator to assess a competency and the relevance on the criteria to measure the command level. A questionnaire in excel (Appendix B) consisting of 4‐point quantitative Likert scale, (1: poorly appropriate, 4: highly appropriate), was used to avoid a neutral answer.

#### 2.3.3 Statistical analysis

The statistical analysis was performed using SPSS 26 (SPSS Inc.). A descriptive analysis of the final BT grades and rubric criteria was performed. Data were reported as mean (±standard error of the mean). Relationships between student perception of the rubrics and academic performance were assessed using bivariate Pearson correlations. Significant correlations (*p* < 0.05) were classified as weak (0.2 < *r* < 0.5), moderate (0.5 < *r* < 0.8), or strong (*r* > 0.8) (O´Rouke et al., 2005).

The content validity analysis was carried out following the Polit and Beck (2006) methodology. The content validity index (suitable validity I‐CVI ≥ 0.78) was calculated for each assessment criteria and descriptors. The intraclass correlation coefficient (ICC) based on a mean rater measurement, absolute agreement, and 2‐way random‐effects model was calculated between the teacher's and students’ marks. The following classification of ICC values was used (Weir, 2005): values 1.00–0.81 (excellent reliability), 0.80–0.61 (very good), 0.60–0.41 (good), 0.40–0.21 (reasonable), and 0.20–0.00 (poor) (González‐Chordá et al., 2016).

## 3 RESULTS AND DISCUSSION

### 3.1 Students’ perception of rubrics

The perception of the students regarding the BT rubrics used by teachers for the evaluation of their final BT is shown in Table 2. The ratings exceed the numerical midpoint of the response scale (5.0) on all items, hence indicating a moderate agreement with the presented items depicting a positive perception of rubrics.

**TABLE 2:** Mean ± standard error of the mean of the students´ perception of the bachelor's theses (BT) rubric.

Item	Mean ± s.e.^a^
1. You are familiar with the rubric that has been used to evaluate your BT	2.60 ± 0.11
2. The rubric accurately assesses the quality of my BT.	3.80 ± 0.16
3. Understanding the rubric has helped me understand the criteria for achieving a good grade.	3.90 ± 0.17
4. The grade you have been given using rubrics matches the one you would give to yourself	4.43 ± 0.20

*Note*: Likert scale (1: Strongly Disagree; 2: Disagree; 3: Neither agree nor disagree; 4: Agree; 5: Strongly Agree).

^a^ s.e.: Standard error of the mean.

The lowest score was for the item “You are familiar with the rubric that has been used to evaluate your BT.” The level of familiarity of students with the rubrics reached only 2.60 points. Familiarity implies that students understand why and how rubrics are used to score their work (Aldukhayel, 2017). Therefore, students involved in this research had only a moderate level of knowledge about rubrics. This suggests that despite the efforts by the faculty to make the BT rubrics known, the students were not as familiar with them as it would be necessary, considering that according to Ferrer‐Pardo et al. (2022), the use of rubrics has demonstrated a higher quality of learning. According to these results, more efforts should be made by teachers, school management team, and degree coordinators to help and encourage students to know the BT rubrics.

Despite the moderate familiarity of students with the rubrics, they mostly agreed with the premise that the rubrics are an accurate tool to assess the quality of BT, as the value reached almost four points on the Likert scale (Table 2). Coincidentally, the students agreed that understanding the criteria of the rubrics has helped them to achieve a good grade (3.90 points). These results basically align with those found by Schamber and Mahoney (2006), who reported that students found the rubric useful for clarifying expectations regarding their final marks of academic projects.

### 3.2 Level of agreement between marks

**FIGURE 1:**
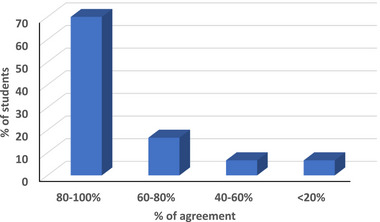
Percentage of agreement between student self‐assessment and the professor mark of the bachelor´s thesis.

Regarding the matching between self‐given grades and the marks given by the teacher committee, it can be observed in Table 2, that this item reached values of 4.43, meaning that in general, students agreed with the obtained qualification. In this sense, when asked for the level of agreement with the teacher mark, up to a 70% of students reported values of 80%–100%, a 17% of students reported values of 60%–80% of agreement, and almost a 7% showed a 40%–60% and less than a 20% of agreement with the final mark of their BT (Figure 1).

According to these results, it seems that the teacher's marks and the student's marks mostly align. Average final BT grades reached up to 7.87 ± 0.08 (Table 3) whereas average self‐given grade was 9.08 ± 0.12. In fact, ICC between marks given by the teachers and marks from self‐evaluation by students was 0.484 (with an inferior and superior limit of 0.121 and 0.669, respectively) which, according to Weir (2005), indicates a good level of congruence between both grades. The agreement between grades has also been reported by other authors such as Sankaran et al. (2023) and Mohiuddin et al. (2019) in higher education. This result supports the idea that the rubrics developed and used were valid as BT assessment tool in this area of science.

**TABLE 3:** Mean and standard error of the mean for each of the rubric criteria of the students assessed.

Rubric criteria	Mean ± s.e.^a^
A. Written expression	7.54 ± 0.16
B. Methodology	9.00 ± 0.62
C. Results, discussion, and conclusions	5.36 ± 0.29
D. Bibliography	8.77 ± 0.25
E. Oral presentation	8.62 ± 0.15
F. Defense	8.05 ± 0.18
Final BT grade	7.87 ± 0.08

^a^ s.e.: Standard error of the mean.

Nevertheless, it should be noted that these results also point to a trend among students to overestimate the quality of their works when compared to the evaluations provided by the teachers. This issue aligns with similar observations in previous studies (García‐Ros, 2011; González‐Chordá et al., 2016; Ferrer‐Pardo et al., 2022). Kruger and Dunning (1999) delved into what causes that overestimation and they linked that overestimation to deficits in metacognitive skills, or the capacity to distinguish accuracy from error.

### 3.3 Relationship between academic results and rubric perception by students

Table 3 shows the average value of the final grades of the BTs as well as the means of the marks for each of the criteria conforming the rubrics.

It should be highlighted that the final grades granted by the members of the thesis committee did not show major differences, thus suggesting a priori the validity and objectivity of the assessment. A possible explanation for this could be the similar background of the teachers of the committee, being all of them full‐time teachers with a similar teaching and research experience. Homogenization of committees is an important parameter for assuring the usefulness of rubrics, as has been reported by González‐Chordá et al. (2016).

Table 4 shows Pearson's correlation between rubric perception and academic performance of students. Understanding the connection between students’ performance and their perception of the validity of rubrics is crucial (Verano‐Tacoronte et al., 2016). It has been suggested that incorporating rubrics enhances the educational process by employing innovative and alternative evaluation methods, while also maintaining consistency and precision (Jönsson and Panadero, 2017). Nevertheless, in this study, no significant relationship has been found between the positive perception of rubric´s validity and the final grades of BTs (Table 4).

**TABLE 4:** Pearson's correlation between rubric perception and academic performance of students (*p‐*value is shown between brackets).

Item	Final Mark of BT
1. You are familiar with the rubric that has been used to evaluate your BT	0.180 (0.379)
2. The rubric accurately assesses the quality of my BT	0.247 (0.224)
3. Understanding the rubric has helped me understand the criteria for achieving a good grade	0,128 (0.532)
4. The grade you have been given matches the one you would give to yourself	0.488^a^ (0.012)

Abbreviation: BT, bachelor's theses.

^a^ Significant levels: *p* < 0.05.

An opposite result would be expected as some authors have reported that the rubrics are not only used to assess but also to teach/learn how to perform a task properly (Panadero and Romero, 2014). The lack of relationship between these parameters could be ascribed to the small variability between marks, as shown in Table 3, so that stronger correlations could not exist (Bennett, 1987). The lack of relationship between the perception of validity of the rubric and academic performance has also been found in other studies focused on oral presentations (Ferrer‐Pardo et al., 2022). Coincidentally, according to Panadero et al. (2023), sometimes rubrics do not contribute to positive gains of performance of students. This aspect is not well clarified here, and this relationship could be recommended as a future direction of research.

### 3.4 Teacher's perception of rubrics and validity of rubrics

Content Validity includes gathering evidence to demonstrate that the assessment content fairly and adequately represents a defined domain of knowledge or performance (Rubio et al., 2003).

Two different approaches have been carried out in the present study to assess the validity of the developed BT rubrics. On the one hand, the perception and opinion of teachers regarding the clarity, suitability, and relevance of the different criteria have been gathered through a questionnaire. Mean and standard errors of the mean of these results are shown in Table 5. On the other hand, those numerical scores have also been used to calculate the content validity index (I‐CVI), which values are reported in Table 6.

**TABLE 5:** Mean ± standard error of the mean of professors´ perception of rubric.

Criteria	Clarity	Suitability	Relevance
A. Written expression	3.95 ± 0.05^1^	3.85 ± 0.06^2^	3.79 ± 0.07^1^
B. Methodology	3.65 ± 0.14^4^	3.85 ± 0.07^2^	3.67 ± 0.15^2^
C. Results, discussion, and conclusions	3.80 ± 0.06^2^	3.93 ± 0.03^1^	3.64 ± 0.14^2^
D. Bibliography	3.75 ± 0.10^3^	3.88 ± 0.06^2^	3.52 ± 0.13^3^
E. Oral presentation	3.79 ± 0.07^3^	3.49 ± 0.14^4^	3.38 ± 0.14^4^
F. Defense	3.82 ± 0.08^2^	3.90 ± 0.06^1^	3.58 ± 0.18^3^
Total	3.78 ± 0.05^3^	3.78 ± 0.05^3^	3.58 ± 0.11^3^

*Note*: Likert scale, (1: poorly appropriate, 4: highly appropriate); Clarity: level of clarity in the wording; Suitability: level of the suitability of the indicator to assess a competency; Relevance: level of relevance on the criteria to measure the command. ^1–2–3–4^, Values with different numbers within the column are significantly different (*p* < 0.05).

**TABLE 6:** Content validity index (I‐CVI) for clarity, suitability, and relevance of each criterion of the rubric.

	I‐CVI		
Rubrics and criteria	Clarity	Suitability	Relevance
A. Written expression			
1. The structure and organization of the work are clear, orderly and comply with instructions	0.84	0.88	0.84
2. The writing is coherent and correct, without spelling or grammatical errors	0.88	0.84	0.80
3. Schemes, figures, tables, etc. are necessary, clarify the text and adhere to the published writing instructions	0.84	0.88	0.80
**B. Methodology**			
1. The materials used are explained thoroughly, correctly, and precisely	0.72	0.80	0.78
2. The research method and materials are explained completely, correctly, and precisely	0.72	0.88	0.80
**C. Results, discussion, and conclusions**			
1. The results are written clearly	0.80	0.92	0.88
2. The results are compared with an adequate number of studies, and they are discussed coherently	0.80	0.92	0.83
3. The conclusions are written clearly, the limitations of the work are indicated, and a forward‐looking perspective is included	0.84	0.84	0.84
4. Conclusions and adequate and pertinent	0.80	0.96	0.80
**D. Bibliography**			
1. All used sources are included	0.80	0.84	0.81
2. All necessary references are included in sufficient numbers, are up‐to‐date, and correctly written	0.68	0.88	0.82
**E. Oral presentation**			
1. The student presents confidently and addresses the tribunal directly	0.88	0.84	0.80
2. The student maintains the attention of the tribunal, handling the content with fluency, and conveys enthusiasm about the topic	0.88	0.64	0.64
3. The font type and size are appropriate and easily readable	0.84	0.64	0.56
4. The colors make the presentation visually appealing and creative	0.78	0.64	0.48
5. The visual elements used are of high quality and enhance the audience's interest	0.78	0.60	0.48
**F.‐ Defense**			
1. Answers all questions posed about the topic	0.84	0.96	0.81
2. Demonstrates confidence in responding to the questions, showcasing a high mastery of the subject	0.84	0.88	0.83

Regarding Table 5, presenting results for clarity, suitability, and relevance of criteria, it can be noticed that, in general, the used rubrics for BT evaluation were highly valued by teachers, values ranging from 3.38 to 3.95. The clarity in the wording of the different criteria and sub‐criteria of the rubric was high. The highest valued criterion was “Written expression,” with 3.95 points, and there were no relevant suggestions to improve this aspect from the teachers. On the other hand, the wording of the “Methodology” criteria was punctuated with the lowest value (3.65). In the responses, teachers suggested that indicating the difficulty of the methodologies and their suitability to the objectives could be included as a criterion. In the case of valuing the suitability of the criteria to assess a competency, “results,” “discussion,” “conclusions,” and “defense” were the best considered, and the worst rated were the criteria for “oral presentation.”

Finally, regarding the relevance of criteria, the highest value was reported for the relevance of “Written expression” (3.79), and the lowest for “Oral presentation” (3.58). This could be related to the fact that, in some way, the teachers value that the oral presentation is a moment of tension for the student, whereas in the written work, there is no such a pressure, and the full potential of the work is shown. Some teachers have reflected this in their answers.

The rubric of the BT, as has been mentioned, included 6 criteria and 11 sub‐criteria with 4‐score ratings (Very appropriate, To improve, Appropriate, and Inadequate). Table 6 shows the content validity index (I‐CVI) for clarity, suitability, and relevance of each criterion of the rubric. Other coefficients have been used in literature to explore the validity of rubrics, such as Aiken´s V coefficient (Sánchez‐Ramírez et al., 2022), Delphi method (Allen & Knight, 2009), and kappa coefficient (García‐Ros, 2011). To avoid the weakness some of these methods have shown (González‐Chordá et al., 2016), a specific method of content validity has been used (Polit & Beck, 2006).

According to Polit and Beck (2006), the values for appropriate validity (I‐CVI) should be higher than 0.78 to be considered acceptable for the validity measurement criteria. Regarding clarity, only “The materials used are explained thoroughly, correctly, and precisely” and “The research method and materials are explained completely, correctly, and precisely” and “All necessary references are included in sufficient numbers, are up‐to‐date, and correctly written,” do not reach 0.78, reaching up to 0.72, 0.72 and 0,68. Hence, the rubric can be considered valid in terms of clarity of criteria, which is a good result and this could be considered a strength of the study.

Regarding suitability of the indicator to assess a competency, only some sub‐criteria related to oral presentation do not reach the minimum level for I‐CVI, such as “The student maintains the attention of the tribunal, handling the content with fluency, and conveys enthusiasm about the topic,” “The font type and size are appropriate and easily readable,” “The colors make the presentation visually appealing and creative,” and “The visual elements used are of high quality and enhance the audience's interest.” In fact, most of the comments of the teachers participating indicated that there are too many criteria to evaluate “Oral presentation.” In general, they proposed adding E1 and E2, pointing out the importance of “expressing oneself clearly and with appropriate scientific or technical language” and E4 and E5 as well, as they considered color part of the visual characteristics that enhance the esthetics of the presentation and enhance the audience's interest. Finally, as far as relevance on the criteria to measure the specific competence, Table 6 shows that, again, the Oral Presentation criteria did not reach values of 0.78, hence not reaching the desired validity. To avoid this weakness, rubrics should be improved in criteria describing Oral Presentation and comments should be considered for further uses of the BT rubric.

Finally, a very good congruence was observed between the teacher's mark using the rubric as a tool and their given mark without the use of the rubric (ICC = 0.78). This value is considered “very good congruence,” according to González‐Chordá et al. (2016).

Considering these results, it can be concluded that the rubrics used for BT assessment in the academic field of food science are valid, that is, those are suitable to measure the progress, evolution, and acquisition of competences by the students according to the European Higher Education Area (EHEA) learning process.

## 4 CONCLUSION

A precise assessment of a food science BT requires reliable and valid tools for objective evaluation. In this study, the validation of a BT assessment system based on a rubric has been achieved. This system may be transferred to other Spanish or abroad Food Science and Technology Degrees as it has been proved to evaluate the validity of a rubric capable of measuring the progress, evolution, and acquisition of competences by the students according to the European Higher Education Area (EHEA) learning process. Future research could explore the correlation between rubric validity perception and students' academic performance.

## AUTHOR CONTRIBUTIONS

**Ana I. Andrés**: Conceptualization; writing—original draft; methodology; validation; visualization; writing—review and editing; software; formal analysis; project administration; data curation; supervision; and resources. **María L. Timón**: Methodology; investigation; writing—original draft; supervision; and resources. **Juan Morillo and Concepción Marín**: Methodology; investigation; resources; and supervision. **Juan Florencio Tejeda**: Investigation; methodology; and resources. **Concepción Ayuso**: Methodology; conceptualization; investigation; writing—review and editing; resources; and supervision.

## CONFLICT OF INTEREST STATEMENT

Declaration of interests: The authors declare that they have no known conflicts of interests or personal relationships that could have appeared to influence the work reported in this paper.

The authors declare the following financial interests/personal relationships that may be considered potential competing interests: Ana Isabel Andrés Nieto.

## Supporting information



Supporting Information

Supporting Information
