# Supplementary material for: Validation of rubric‐based evaluation for bachelor's theses in a food science and technology degree
Source: J Food Sci. 2024 Apr 5;89(5):3129–38. doi: 10.1111/1750-3841.17044 (PMC13281131; doi:10.1111/1750-3841.17044)
Supplement: Supplementary file 1 — Supporting Information [file JFDS-89-3129-s002.docx]

Appendix A.- Rubrics used of BT assessment in the Degree of Food Science and Technology.

| **A.-WRITTEN EXPRESSION AND WRITTEN PRESENTATION OF THE WORK (15%)** | | | | | | | |
| --- | --- | --- | --- | --- | --- | --- | --- |
|  | **Very appropriate** | **To improve** | | **Appropriate** | | **Inadequate** | |
| **Organization and structure (Score from 0 to 10)** | Organization and structure are clear and suitable | | Organization and structure are clear and adequate, but some non-essential sections are missing | | Organization and structure are clear and adequate, but some essential sections are missing | | Organization and structure are incoherent and disjointed Essential parts of the work are missing |
| **Writing (Score from 0 to 10)** | Coherent and correct writing, with no spelling mistakes or grammatical errors | | Coherent and correct writing for the most part, with no spelling mistakes or grammatical errors | | Inconsistent and incorrect wording. No spelling mistakes or grammatical errors | | Inconsistent and incorrect writing, with spelling mistakes and/or grammatical errors |
| **Diagrams, tables, graphs (Score 0 to 10)** | Complies exactly with published drafting standards | | Complies with most of the published drafting standards. | | Complies with some published drafting standards | | Does not conform to published drafting standards |
| **Objectives (Score from 0 to 10)** | Objectives are clear and appropriate, written in the infinitive form | | Objectives are adequate and written in the infinitive although they could have been made more explicit | | Objectives are not adequate, although they are well written and explained | | Objectives are not adequate, neither well written nor explained |
| **B.-METHODOLOGY (20%)** | | | | | | | |
|  | **Very appropriate** | | **To improve** | | **Appropriate** | | **Inadequate** |
| **Methodology presentation (Score from 0 to 10)** | The research methodology and materials are fully, correctly and concretely explained | | The research methodology and materials are explained fully, correctly, but could have been explained more concretely | | The research methodology and materials are explained incompletely, incorrectly or unclearly | | The research methodology and materials used are not explained |
| **C.-ADEQUACY OF RESULTS, DISCUSSION AND CONCLUSIONS (20%)** | | | | | | | |
|  | **Very appropriate** | | **To improve** | | **Appropriate** | | **Inadequate** |
| **Results and discussion (Score from 0 to 10)** | The results are clearly written. They are compared with other studies and discussed in a coherent way | | Results are mostly clearly written. Comparisons with other studies are made, but not sufficiently so. Consistently discussed | | Results are mostly clearly written. Not compared with other studies. Not consistently discussed | | Results are not clearly written. They are not compared with other studies. They are not consistently discussed |
| **Conclusions**  **( Score from 0 to 10)** | They are absolutely congruent with the topic addressed. They are clearly written. Limitations of the work - if any - are indicated. The prospective of the work is included | | They are congruent with the topic addressed. They are clearly written. The limitations of the work - if any - are not stated. Prospective work is not included | | They are not entirely congruent with the topic addressed. They are written in a way that could be improved. The limitations of the work, if any, are not indicated. The prospective of the work is not included | | They are not congruent with the topic addressed. They are incorrectly written. The limitations of the work, if any, are not indicated. Prospective work is not included |
| **D.-BIBLIOGRAPHY (15%)** | | | | | | | |
|  | **Very appropriate** | | **To improve** | | **Appropriate** | | **Inadequate** |
| **Sources and references (Score 0 to 10)** | All sources are included, all references are included, match the text and are up-to-date and correctly worded | | Almost all sources are included, almost all references match the text and are up to date | | Almost all sources are included, almost all references are included (but can be completed), they are not up to date and there are some flaws in the wording | | A large majority of sources and references are not included, do not coincide with the text and are not up to date. They are not correctly written |
| **E.-ORAL PRESENTATION (15%)** | | | | | | | |
|  | **Very appropriate** | | **To improve** | | **Appropriate** | | **Inadequate** |
| **Attitude and speech (Score from 0 to 10)** | The student delivers a confident presentation, addressing the panel, maintaining their attention and handling transparencies or other media with ease. Conveys enthusiasm for the subject | | The student follows the thread of the slides without the need to read them verbatim and addresses the examiner. The volume of voice used in the presentation is appropriate. Sometimes generates interest and enthusiasm for the topic | | The student follows the thread of the slides but presents the content literally. The volume of voice used in the presentation is adequate. Attempts to generate interest or enthusiasm | | The presentation jumps abruptly between slides and loses the thread of the presentation. The volume of voice used is too low to be perceived clearly. Does not generate much interest or enthusiasm |
| **Aesthetics of the presentation (Score from 0 to 10)** | Font size and typeface are appropriate and read clearly. Colors make the presentation pleasing to the eye and creative. Quality visual elements, which increase the interest of the listener | | Font size and typeface can be improved but are clearly readable. Colors make the presentation pleasing to the eye and creative. Visual elements are correct | | Font size and typeface can be improved and are not clearly readable. Colors make the presentation pleasing to the eye and creative. Poor visual elements. | | Font size and typeface are inadequate and do not read clearly. Colors make the presentation unpleasant. No visual elements are included. |
| **F.-DEFENCE (15%)** | | | | | | | |
|  | **Very appropriate** | | **To improve** | | **Appropriate** | | **Inadequate** |
| **Answers to questions (Score from 0 to 10)** | Answers all the questions asked on the topic accurately | | Answers most of the questions posed on the topic accurately | | Answers few questions accurately | | Cannot answer the questions asked |
