# Supplementary material for: Validation of rubric‐based evaluation for bachelor's theses in a food science and technology degree
Source: J Food Sci. 2024 Apr 5;89(5):3129–38. doi: 10.1111/1750-3841.17044 (PMC13281131; doi:10.1111/1750-3841.17044)
Supplement: Supplementary file 2 — Supporting Information [file JFDS-89-3129-s001.docx]

*Appendix B.- Excel questionnaire given to the teachers, to evaluate the clarity, suitability and relevance of each of the criteria of the rubrics.*

| **Estimado evaluador:  Agradeceríamos que evaluaras cada uno de estos criterios marcando con una X en la casilla que consideres oportuna según estos tres parámetros: Claridad, Idoneidad, Relevancia** | | | | | | | | | | | | | | | |
| --- | --- | --- | --- | --- | --- | --- | --- | --- | --- | --- | --- | --- | --- | --- | --- |
|  |  |  | **Claridad en la formulación de los criterios** | | | | **Idoneidad del criterio para evaluar el indicador marcado** | | | | **Relevancia o grado de adecuación del criterio con el indicador** | | | |  |
|  |  |  | **1: menos adecuado y 4: más adecuado** | | | | | | | | | | | |  |
|  |  |  |  |  |  |  |  |  |  |  |  |  |  |  |  |
|  |  | **Competencias para la empleabilidad (o soft skills)*** analizadas: resolución de problemas, trabajo en equipo, toma de decisiones, capacidad de adaptación, creatividad, comunicación y liderazgo | | | | | | | | | | | | | |
| *Entendidas también como habilidades o competencias transversales para los profesionales del Siglo XXI | | | | | | | | | | | | | | | |
|  |  |  |  |  |  |  |  |  |  |  |  |  |  |  |  |
|  |  | **Por favor, evalúa, marcando con una X cada indicador** |  |  |  |  |  |  |  |  |  |  |  |  |  |
| **Resolución de problemas** | | | **Claridad** | | | | **Idoneidad** | | | | **Relevancia** | | | | **Comentarios** |
|  |  |  | 1 | **2** | **3** | **4** | **1** | **2** | **3** | **4** | **1** | **2** | **3** | **4** |  |
| **Nivel** |  | **Indicadores** |  |  |  |  |  |  |  |  |  |  |  |  |  |
| A | i1 | Priorizo y me centro únicamente en aquellos aspectos que me afectan directamente. |  |  |  |  |  |  |  |  |  |  |  |  |  |
|  | i2 | Identifico los problemas y los analizo. |  |  |  |  |  |  |  |  |  |  |  |  |  |
|  |  |  |  |  |  |  |  |  |  |  |  |  |  |  |  |
| B | i3 | Conozco la problemática y adopto decisiones acertadas y justificadas ante imprevistos y dificultades, persiguiendo la consecución de objetivos concretos. |  |  |  |  |  |  |  |  |  |  |  |  |  |
|  | i4 | Busco soluciones pensando en el camino a seguir. |  |  |  |  |  |  |  |  |  |  |  |  |  |
|  |  |  |  |  |  |  |  |  |  |  |  |  |  |  |  |
| C | i5 | Identifico y anticipo problemas, los abordo y aporto nuevas vías de solución. |  |  |  |  |  |  |  |  |  |  |  |  |  |
|  | i6 | Me desenvuelvo con soltura ante situaciones díficiles. |  |  |  |  |  |  |  |  |  |  |  |  |  |
|  | i7 | No me conformo con el cumplimiento de mis propias funciones sino que aplico nuevas ideas y fórmulas en los procesos con resultados efectivos. |  |  |  |  |  |  |  |  |  |  |  |  |  |
|  |  |  |  |  |  |  |  |  |  |  |  |  |  |  |  |
| **Trabajo en Equipo** | | | **Claridad** | | | | **Idoneidad** | | | | **Relevancia** | | | | **Comentarios** |
|  |  |  | **1** | **2** | **3** | **4** | **1** | **2** | **3** | **4** | **1** | **2** | **3** | **4** |  |
| **Nivel** |  | **Indicadores** |  |  |  |  |  |  |  |  |  |  |  |  |  |
| A | i8 | Aporto nuevas ideas y pensamientos al grupo. |  |  |  |  |  |  |  |  |  |  |  |  |  |
|  | i9 | Colaboro con el resto del equipo con el fin de lograr objetivos comunes. |  |  |  |  |  |  |  |  |  |  |  |  |  |
|  | i10 | Comparto la información necesaria con el grupo para realizar las tareas. |  |  |  |  |  |  |  |  |  |  |  |  |  |
|  |  |  |  |  |  |  |  |  |  |  |  |  |  |  |  |
| B | i11 | Ofrezco ayuda a los demás miembros de mi grupo cuando veo que están sobrecargados. |  |  |  |  |  |  |  |  |  |  |  |  |  |
|  | i12 | Me implico en los debates de clase, preparándolos, aportando ideas, respetando las opiniones e ideas de mis compañeros... |  |  |  |  |  |  |  |  |  |  |  |  |  |
|  | i13 | Tengo una visión global de los objetivos del equipo y soy capaz de renunciar a los objetivos personales en beneficio de los objetivos grupales. |  |  |  |  |  |  |  |  |  |  |  |  |  |
|  | i14 | Hago lo que el resto del grupo espera de mí en el tiempo indicado y de calidad. |  |  |  |  |  |  |  |  |  |  |  |  |  |
|  |  |  |  |  |  |  |  |  |  |  |  |  |  |  |  |
| C | i15 | Animo y motivo a mis compañeros reconociendo su mérito. |  |  |  |  |  |  |  |  |  |  |  |  |  |
|  | i16 | Adopto diferentes roles dentro de mi equipo (por ejemplo, llevando a cabo diferentes tareas en función de la actividad). |  |  |  |  |  |  |  |  |  |  |  |  |  |
|  | i17 | Actúo de forma conciliadora cuando surgen diferencias de opiniones entre los miembros del equipo. |  |  |  |  |  |  |  |  |  |  |  |  |  |
|  | i18 | Comparto tanto el éxito como el fracaso en el trabajo en equipo. |  |  |  |  |  |  |  |  |  |  |  |  |  |
|  | i19 | Promuevo la cooperación con otros equipos y comparto información con ellos. |  |  |  |  |  |  |  |  |  |  |  |  |  |
|  |  |  |  |  |  |  |  |  |  |  |  |  |  |  |  |
| **Capacidad de adaptación / flexibilidad** | | | **Claridad** | | | | **Idoneidad** | | | | **Relevancia** | | | | **Comentarios** |
|  |  |  | **1** | **2** | **3** | **4** | **1** | **2** | **3** | **4** | **1** | **2** | **3** | **4** |  |
| **Nivel** |  | **Indicadores** |  |  |  |  |  |  |  |  |  |  |  |  |  |
| A | i20 | Cuando quiero conseguir un resultado analizo varias opciones. |  |  |  |  |  |  |  |  |  |  |  |  |  |
|  | i21 | Me resulta fácil comprender a las personas, ponerme en su lugar y adaptarme a varios perfiles. |  |  |  |  |  |  |  |  |  |  |  |  |  |
|  | i22 | Reconozco los cambios y los asumo como un reto. |  |  |  |  |  |  |  |  |  |  |  |  |  |
|  |  |  |  |  |  |  |  |  |  |  |  |  |  |  |  |
| B | i23 | Me resulta fácil cambiar la forma de trabajar para alcanzar mis objetivos como alumno. |  |  |  |  |  |  |  |  |  |  |  |  |  |
|  | i24 | Puedo analizar y desarollar varias alternativas y soluciones para distintos problemas. |  |  |  |  |  |  |  |  |  |  |  |  |  |
|  |  |  |  |  |  |  |  |  |  |  |  |  |  |  |  |
| C | i25 | En situaciones más dificiles puedo cambiar rápido mi estrategia o mi forma de actuar. |  |  |  |  |  |  |  |  |  |  |  |  |  |
|  | i26 | Impulso el cambio en varias actividades y puedo desarollar nuevos enfoques integrando opiniones internas y externas. |  |  |  |  |  |  |  |  |  |  |  |  |  |
|  |  |  |  |  |  |  |  |  |  |  |  |  |  |  |  |
| **Comunicación** | | | **Claridad** | | | | **Idoneidad** | | | | **Relevancia** | | | | **Comentarios** |
|  |  |  | **1** | **2** | **3** | **4** | **1** | **2** | **3** | **4** | **1** | **2** | **3** | **4** |  |
| **Nivel** |  | **Indicadores** |  |  |  |  |  |  |  |  |  |  |  |  |  |
| A | i27 | Soy capaz de emitir mensajes claros y ordenados. |  |  |  |  |  |  |  |  |  |  |  |  |  |
|  | i28 | Estructuro los mensajes de forma lógica. |  |  |  |  |  |  |  |  |  |  |  |  |  |
|  | i29 | Sé atraer y mantener la atención de mis compañeros o profesores. |  |  |  |  |  |  |  |  |  |  |  |  |  |
|  |  |  |  |  |  |  |  |  |  |  |  |  |  |  |  |
| B | i30 | Me esfuerzo por escuchar a los demás y me aseguro de comprenderlos correctamente. |  |  |  |  |  |  |  |  |  |  |  |  |  |
|  | i31 | Soy capaz de transmitir bien ideas, informaciones o instrucciones complejas. |  |  |  |  |  |  |  |  |  |  |  |  |  |
|  | i32 | Compruebo si he sido entendido correctamente por el interlocutor. |  |  |  |  |  |  |  |  |  |  |  |  |  |
|  | i33 | Dedico el tiempo adecuado en mis exposiciones. |  |  |  |  |  |  |  |  |  |  |  |  |  |
|  | i34 | Escojo el medio y las formas más adecuadas para comunicarme de acuerdo con la situación, el mensaje y el receptor. |  |  |  |  |  |  |  |  |  |  |  |  |  |
|  |  |  |  |  |  |  |  |  |  |  |  |  |  |  |  |
| C | i35 | Recibo con agrado la opinión o feedback de mis compañeros o profesores y la tengo en cuenta para preparar mis próximas exposiciones. |  |  |  |  |  |  |  |  |  |  |  |  |  |
|  | i36 | Realizo presentaciones en público de alto impacto. |  |  |  |  |  |  |  |  |  |  |  |  |  |
|  | i37 | Soy capaz de adaptar mi estilo de presentar a personas de diversas culturas y países. |  |  |  |  |  |  |  |  |  |  |  |  |  |
|  | i38 | Consigo convencer a los demás a partir de mi discurso, propuesta o proyecto. |  |  |  |  |  |  |  |  |  |  |  |  |  |
|  |  |  |  |  |  |  |  |  |  |  |  |  |  |  |  |
| **Creatividad** | | | **Claridad** | | | | **Idoneidad** | | | | **Relevancia** | | | | **Comentarios** |
|  |  |  | **1** | **2** | **3** | **4** | **1** | **2** | **3** | **4** | **1** | **2** | **3** | **4** |  |
| **Nivel** |  | **Indicadores** |  |  |  |  |  |  |  |  |  |  |  |  |  |
| A | i39 | Siempre tengo curiosidad e interes por descubrir y aprender cosas nuevas. |  |  |  |  |  |  |  |  |  |  |  |  |  |
|  | i40 | Puedo visualizar las situaciones desde distintos puntos de vista. |  |  |  |  |  |  |  |  |  |  |  |  |  |
|  | i41 | Analizo mis propias ideas con el objetivo de maximizar y mejorar los resultados. |  |  |  |  |  |  |  |  |  |  |  |  |  |
|  |  |  |  |  |  |  |  |  |  |  |  |  |  |  |  |
| B | i42 | Utilizo distintas tecnicas para crear ideas (ej. lluvia de ideas, generando preguntas, asociando...). |  |  |  |  |  |  |  |  |  |  |  |  |  |
|  | i43 | Con poca información o poco material puedo crear con facilidad nuevas ideas u objetos. |  |  |  |  |  |  |  |  |  |  |  |  |  |
|  |  |  |  |  |  |  |  |  |  |  |  |  |  |  |  |
| C | i44 | Demuestro originalidad e inventiva en los trabajos individuales o de grupo. |  |  |  |  |  |  |  |  |  |  |  |  |  |
|  | i45 | Me gusta y puedo implementar con facilidad nuevas ideas o nuevos proyectos. |  |  |  |  |  |  |  |  |  |  |  |  |  |
|  | i46 | Soy capaz de redefinir ideas, conceptos u objetos de manera diferente a como se había hecho hasta entonces aprovechándolos para fines completamente nuevos. |  |  |  |  |  |  |  |  |  |  |  |  |  |
|  |  |  |  |  |  |  |  |  |  |  |  |  |  |  |  |
| **Liderazgo** | | | **Claridad** | | | | **Idoneidad** | | | | **Relevancia** | | | | **Comentarios** |
|  |  |  | **1** | **2** | **3** | **4** | **1** | **2** | **3** | **4** | **1** | **2** | **3** | **4** |  |
| **Nivel** |  | **Indicadores** |  |  |  |  |  |  |  |  |  |  |  |  |  |
| A | i47 | Me aseguro de que los miembros de mi equipo trabajan con las directrices adecuadas. |  |  |  |  |  |  |  |  |  |  |  |  |  |
|  | i48 | Conozco las capacidades/conocimientos de mis compañeros y les asigno objetivos/responsabilidades en función de los mismos. |  |  |  |  |  |  |  |  |  |  |  |  |  |
|  | i49 | Informo a mis compañeros de las decisiones que les pueden afectar. |  |  |  |  |  |  |  |  |  |  |  |  |  |
|  | i50 | Creo un ambiente positivo en el grupo. |  |  |  |  |  |  |  |  |  |  |  |  |  |
|  |  |  |  |  |  |  |  |  |  |  |  |  |  |  |  |
| B | i51 | Delego en función de las habilidades de los miembros de mi equipo. |  |  |  |  |  |  |  |  |  |  |  |  |  |
|  | i52 | Doy feedback tanto positivo como negativo a mis compañeros. |  |  |  |  |  |  |  |  |  |  |  |  |  |
|  | i53 | Facilito la participación de mis compañeros en la toma de decisiones y desarollo un ambiente de cooperación. |  |  |  |  |  |  |  |  |  |  |  |  |  |
|  | i54 | Comparto los éxitos con mis compañeros y me hago responsable de las equivocaciones de los mismos. |  |  |  |  |  |  |  |  |  |  |  |  |  |
|  | i55 | Proporciono apoyo, consejo y comunicación adecuados para mejorar el rendimiento. |  |  |  |  |  |  |  |  |  |  |  |  |  |
|  |  |  |  |  |  |  |  |  |  |  |  |  |  |  |  |
| C | i56 | Gestiono con éxito grupos de trabajo complejos y heterógeneos en su grado de madurez. |  |  |  |  |  |  |  |  |  |  |  |  |  |
|  | i57 | Saco a la luz y a tiempo los conflictos del grupo y trabajo con mi equipo para resolverlos. |  |  |  |  |  |  |  |  |  |  |  |  |  |
|  | i58 | Adopto diferentes estilos de mando en función del nivel de madurez de los miembros de mi equipo. |  |  |  |  |  |  |  |  |  |  |  |  |  |
|  | i59 | Hago que se escuchen y respeten las opiniones o posiciones divergentes. |  |  |  |  |  |  |  |  |  |  |  |  |  |
|  | i60 | Consigo compromiso de los diferentes puntos de vista en las decisiones tomadas. |  |  |  |  |  |  |  |  |  |  |  |  |  |
|  |  |  |  |  |  |  |  |  |  |  |  |  |  |  |  |
| **Toma de decisiones** | | | **Claridad** | | | | **Idoneidad** | | | | **Relevancia** | | | | **Comentarios** |
|  |  |  | **1** | **2** | **3** | **4** | **1** | **2** | **3** | **4** | **1** | **2** | **3** | **4** |  |
| **Nivel** |  | **Indicadores** |  |  |  |  |  |  |  |  |  |  |  |  |  |
| A | i61 | Tomo decisiones sólo si es con la consulta previa de un profesor. |  |  |  |  |  |  |  |  |  |  |  |  |  |
|  | i62 | Tomo decisiones sobre cuestiones que ya están en marcha sin cuestionarme los criterios que se han seguido. |  |  |  |  |  |  |  |  |  |  |  |  |  |
|  | i63 | Tomo decisiones motivadas por el factor emocional, mostrándome más impulsivo que racional. |  |  |  |  |  |  |  |  |  |  |  |  |  |
|  |  |  |  |  |  |  |  |  |  |  |  |  |  |  |  |
| B | i64 | Tomo las decisiones justas cuando surgen dificultades o cuando se trata de elegir entre varias alternativas de solución ante un problema. |  |  |  |  |  |  |  |  |  |  |  |  |  |
|  | i65 | Tengo en cuenta las consecuencias positivas y negativas de las distintas alternativas. |  |  |  |  |  |  |  |  |  |  |  |  |  |
|  |  |  |  |  |  |  |  |  |  |  |  |  |  |  |  |
| C | i66 | Me anticipo a los hechos para tomar decisiones. |  |  |  |  |  |  |  |  |  |  |  |  |  |
|  | i67 | Minimizo la carga emotiva que conllevan las decisiones delicadas, a las que llego tras un proceso reflexivo y analítico. |  |  |  |  |  |  |  |  |  |  |  |  |  |
|  | i68 | Elijo la opción que considero más adecuada de una manera justificada. |  |  |  |  |  |  |  |  |  |  |  |  |  |
